# Critical values notification: A nationwide survey of practices among clinical laboratories across Nigeria

**DOI:** 10.4102/ajlm.v12i1.2249

**Published:** 2023-12-15

**Authors:** Lucius C. Imoh, Idris Y. Mohammed, Ifeyinwa D. Nnakenyi, Ephraim U. Egbuagha, Tomisin M. Adaja, Chinelo P. Onyenekwu

**Affiliations:** 1 Department of Chemical Pathology, Faculty of Medical Sciences, University of Jos, Jos, Plateau State, Nigeria; 2 Department of Chemical Pathology & Immunology, College of Health Sciences, Bayero University and Aminu Kano Teaching Hospital, Kano, Kano State, Nigeria; 3 Department of Chemical Pathology, Faculty of Medical Sciences, University of Nigeria Nsukka and University of Nigeria Teaching Hospital, Enugu, Enugu State, Nigeria; 4 Department of Pathology, Clinix Healthcare Ltd, Lagos, Lagos State, Nigeria; 5 Department of Chemical Pathology, Federal Medical Centre, Owo, Ondo State, Nigeria; 6 Department of Chemical Pathology, Ben Carson Snr School of Medicine, Babcock University and Babcock University Teaching Hospital, Ilishan-Remo, Ogun State, Nigeria

**Keywords:** critical values, critical value reporting, critical value notification, post-analytical services, laboratory quality management

## Abstract

**Background:**

Critical value notification (CVN) entails notifying doctors or other laboratory users of aberrant laboratory results that threaten the patient’s life and of any values for which reporting delays could negatively impact the patient’s health. Critical value notification practices in clinical laboratories in Nigeria and sub-Saharan Africa are largely unknown.

**Objective:**

We conducted a nationwide survey to obtain baseline information on CVN practice by Nigeria’s laboratories.

**Methods:**

This cross-sectional study was conducted among purposively selected secondary- and tertiary-tier, public and private clinical laboratories across northern and southern Nigeria between October 2015 and December 2015. Consenting senior laboratory staff completed and returned a structured questionnaire, that gathered data on respondents’ demographics, designations, and institutional characteristics and practices regarding CVN.

**Results:**

One hundred and thirty-four laboratories responded to the questionnaires. Only 69 (51.5 %) laboratories practised CVN; only 23 (33.3%) had existing written policies guiding the practice. Most (43; 62.3%) laboratories use similar critical values (CVs) for adult and paediatric populations. Most laboratories (27; 39.1%) obtained their CVs by combining published literature and local opinions from stakeholders. Physical dispatch (42; 60.9%) followed by telephone calls (38; 55.1%) were the most common means of notification. Private laboratories, compared with public hospital laboratories, were likelier to have separate paediatric CV lists (*p* = 0.019) and practise telephone notifications (*p* < 0.001).

**Conclusion:**

Critical value notification practices vary and are often suboptimal in many clinical laboratories in Nigeria, which is exacerbated by the absence of guiding policies and national recommendations for post-analytical procedures.

**What this study adds:**

This study provides baseline information on CVN practice by Nigeria’s laboratories. The study explores the causes of practice variations that can serve as a foundation for enhancing critical reporting and post-analytical services, particularly in clinical laboratories in sub-Saharan Africa.

## Introduction

Laboratory results are essential for optimal patient care. Prompt release and transmission of crucial test results significantly impacts healthcare decisions and patient outcomes.^1,2,3,4^ A critical value in laboratory practice generally refers to a laboratory result that suggests a potentially life-threatening condition needing prompt and appropriate medical intervention. As defined by Lundberg, the critical value is a result that is so highly abnormal that it is deemed potentially lethal or likely to cause considerable morbidity and necessitates immediate intervention.^5,6^ The expression has been used interchangeably with alternative terms such as ‘panic value’ and ‘alarm value’.^5^

Many laboratories have long focused on improving pre-analytical processes, and the use of automation and improved analytical systems is helping to produce more accurate and timely results.^7^ However, standardisation of post-analytical processes, such as critical value reporting, is more challenging and potentially prone to human mistakes or violations, with untoward implications for patient safety.^7,8^ The importance of timely delivery of critical values (CVs) is underscored by the fact that reporting critical results is a vital quality indicator for the post-analytical phase of the total testing process. Additionally, accreditation and regulatory authorities demand that laboratories have policies in place to communicate critical results to ensure patient safety.^9,10,11,12^

There are few or no established standards or guidelines for reporting CVs in many countries and regions, and crucial results-reporting practices vary widely around the globe.^7,9^ There are many reasons for this heterogeneity in practice, including differences in patient demographics, staffing levels, instrumentation, and institutional structures.^13^ From published literature, some of the areas of considerable differences in CVs reporting practices include the policy framework for critical value notification (CVN), selection of CVs, the source(s) for determining critical limits, acceptable timeframe for delivering CVs, repeat testing for CVs and which results to report.^7,11^ Other issues relating to the communication of CVs include means of reporting, to whom to report, and how to handle cases of unreachable caregivers.^7,14,15,16,17^

Nigeria has no known standard practices for CVN. Any efforts at harmonising CVN would logically begin with assessing current practices and the underlying basis for such practices across laboratories. Therefore, we surveyed CVN for routine clinical chemistry and haematology analytes to obtain baseline information on the practice of CVN in Nigeria.

## Methods

### Ethical considerations

Ethical approval was obtained from the Human Research Ethical Committee of the Jos University Teaching Hospital (JUTH/DCS/ADM/127/XXV/152). The respondents gave their written informed consent, and confidentiality was maintained per the Declaration of Helsinki’s ethical standards for medical research involving human beings. This was done by the allocation of unique and anonymised study codes. The research data were stored electronically on password-protected devices accessible only by the researchers.

### Study design and duration

This cross-sectional nationwide survey was conducted between October 2015 to December 2015 among secondary- and tertiary-tier clinical laboratories. These include laboratories of public hospitals such as general/district secondary care hospitals and laboratories of teaching hospitals that service several peripheral hospitals. The study also involved laboratories of private clinics and hospitals, faith-based health institutions, and commercial laboratories. These laboratories provide routine chemistry tests such as glucose, bilirubin, urea, creatinine, electrolytes, urine, triglycerides, cholesterols, liver enzymes, hormones and tumour markers, as well as basic haematology tests, such as haematocrit, complete blood count and clotting profile. The study involved a total sampling of consenting laboratories in 25 purposively selected states. These states had a large distribution of secondary- and tertiary-tier (including public and private) clinical laboratories across the six geopolitical zones in the north and south of Nigeria. The federal capital territory (Abuja) and 24 of the 36 states in the country were surveyed. A structured questionnaire was given to consenting laboratories and completed by any senior laboratory staff deemed competent by the head of the laboratory to respond to the questionnaires (one person per laboratory). According to the scheme of service, these laboratory staff include pathologists, pathologists-in-training (resident doctors), laboratory directors, and senior laboratory scientists or technologists. The questionnaire gathered data on demographics, respondents’ designations, and the type and speciality of laboratory or health institutions. We also looked into data on the existence of and practices surrounding CVNs, and the handling of CVs. The questionnaire addressed the CVN policy framework, which refers to documented management commitment, processes and procedures for handling CVs in this manuscript.

The responders were invited to select each item applicable to a specific question and provide more information if necessary. The self-administered questionnaire was pre-tested using responses from three laboratory specialists who were knowledgeable about the subject and were not part of the study respondents to ensure the validity and reliability of the collected data. The pre-test explored how well the questionnaire represents a theoretical concept of the study. Areas of the questionnaire that could be misinterpreted were changed or left out of the study’s questions, for instance the clarification of the institution’s designation as government or public versus private or commercial and the level of health institution (primary, secondary, or tertiary). Furthermore, as a result of pre-testing the questionnaire, it became necessary to allow the respondents to select all options that applied to a specific question and to provide additional information whenever necessary. Ambiguous or loosely used laboratory terms were avoided, or a term definition was provided, for instance the definition of CV policy as described above and the term ‘read-back policy’. The respondents were also encouraged to provide a copy of their laboratory’s critical values for sighting.

### Statistical analysis

Microsoft Excel^®^ version 15.0 (Microsoft Corp. 2013, Redmond, Washington, United States) was used to compile the data before being exported for analysis into Statistical Product and Service Solutions version 23.0 software (IBM Corp. 2015, Armonk, New York, United States). Charts, frequency tables, counts, and percentages were used to present descriptive data. The chi-square or Fisher’s exact test was used for inferential statistics to test associations, as appropriate. A *p*-value of 0.05 or lower was considered significant.

## Results

**FIGURE 1:**
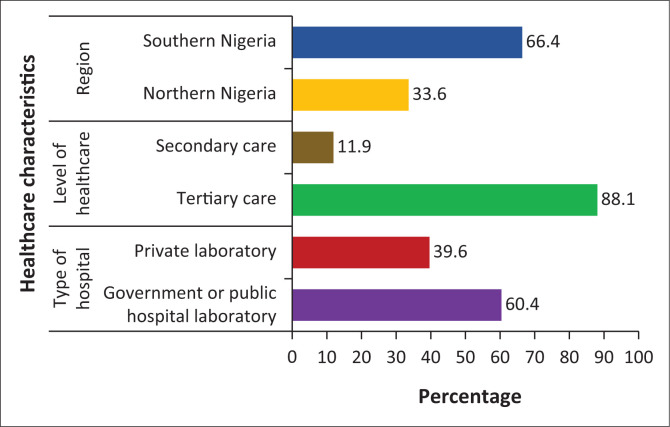
General characteristics of 134 surveyed laboratories across 25 states in Nigeria, October 2015 to December 2015.

One hundred and thirty-four (*n* = 134) laboratories returned their questionnaires from a total of 185 laboratories surveyed, giving a response rate of 72.4%. Forty-five (33.6%) were in northern Nigeria and 89 (66.4%) in southern Nigeria; 81 laboratories (60.4%) served government/public hospitals, and 118 (88.1%) were situated in tertiary centres (Figure 1).

### Critical value notification operating policy and critical value limits list

Sixty-nine laboratories (51.5 %) practise CVN, while 65 (48.5%) do not practise CVN. Among the CVN-practising laboratories, only 23 (33.3%) have an existing written CVN policy (Table 1). Also, the actual list of CV limits was sighted in only 26 (37.7%) laboratories that reported practising CVN. Forty-three (62.3%) laboratories used the same CVs for both adult and paediatric populations. Twenty-seven (39.1%) laboratories obtained their CVs by combining published literature and local opinions from stakeholders, such as clinical and laboratory staff.

**TABLE 1:** Aspects of critical value notification practices in 134 surveyed laboratories across 25 states in Nigeria, October 2015 to December 2015.

Variables	Categories	Frequency	Percentage
Practise CVN (*n* = 134)	Yes	69	51.5
	No	65	48.5
Written CVN policy (*n* = 69)	Yes	23	33.3
	No	46	66.7
Paediatric CV list (*n* = 69)	Yes	26	37.7
	No	43	62.3
Actual CV list (*n* = 69)	Yes	26	37.7
	No	43	62.3
Same adult and paediatric CV list (*n* = 69)	Yes	43	62.3
	No	26	37.7
Sources of CV list (*n* = 69)	Local clinical opinion	7	10.1
	Published literature	18	27.5
	Local clinical opinion and published literature	27	39.1
	Review of laboratory practice	6	8.7
	No response	11	15.9

CV, critical value; CVN, critical value notification.

### Analytical handling of critical values

Among the laboratories that practise CVN, most (*n* = 66; 95.7%) repeated the assay for results with CV, mostly once (37.7%). Nearly three-quarters of the laboratories (*n* = 51; 73.9%) had no policy to repeat tests with CVs. Among the CVN-practising laboratories that repeated testing, 18 (27.3%) laboratories reported the average of the repeated results; 9 (13.6%) laboratories reported the most severe CVs generated, while 8 (12.1%) reported the least severe CVs (Table 2).

**TABLE 2:** Analytical handling of critical values in 69 laboratories practising critical value notification across 25 states in Nigeria, October 2015 to December 2015.

Variables	Categories	Frequency	Percentage
Repeat testing (*n* = 69)	Yes	66	95.7
	No	3	4.3
Frequency of repeat testing (*n* = 69)	1	26	37.7
	2	14	20.3
	3	6	8.7
	Unspecified	23	33.3
Policy for handling repeated results (*n* = 69)	Yes	18	26.1
	No	57	73.9
Value of repeat test to report (*n* = 66)	Average of repeat testing values	18	27.3
	Most critical of the values	9	13.6
	Least critical of the values	8	12.1
	Technologist’s discretion	2	3.0
	No laid down rule	18	27.3
	No response	11	16.7

### Post-analytic management of critical value notification

Senior laboratory staff (pathologists, pathologists in-training, laboratory managers and senior laboratory scientists) take responsibility for notifying critical results in most cases (*n* = 49; 71.0%), while CVN is the responsibility of the technologist on the bench in 32 (46.4%) laboratories (Table 3). The most common means of notification was physical dispatch (*n* = 42; 60.9%), followed by telephone calls (*n* = 38; 55.1%) and short message service (*n* = 8; 12.6%). Nine (23.7%) laboratories with phone call notification practised a read-back policy, while 29 (76.3%) did not. Thirty-four (49.3%) laboratories notified the doctor or nurse directly involved in the patient’s care (Table 3). Only 18 laboratories (26.1%) have mechanisms to deal with situations where the patient’s caregiver is unreachable, while 51 (73.9%) laboratories do not. In 40 (58%) laboratories, there were no specifications on the time limit to notify a caregiver of a CV. Other time limits include: ‘within 10 min’ (*n* = 2; 3.0%), ‘within 30 min’ (*n* = 6; 8.7%), ‘within 60 min’ (*n* = 3; 4.4%), ‘within 24 min’ (*n* = 2; 3.0%), or no response (*n* = 16; 23.2%).

**TABLE 3:** Post-analytic management of critical values in 69 laboratories practising critical value notification across 25 states in Nigeria, October 2015 to December 2015.

Variables	Categories	Frequency†	Percentage
Person(s) responsible for notification of CV (*n* = 69)	Technologist on the bench	32	46.4
	Senior laboratory staff (laboratory manager, pathologists in-training, pathologist)	49	71.0
	Receptionist, pre-analytical section or call centre	1	1.4
Mode of notification (*n* = 69)	Telephone call	38	55.1
	SMS	8	11.6
	Email	0	0.0
	Physical dispatch	43	62.3
Person(s) to receive notification (*n* = 69)	The person who ordered the test	23	33.3
	Doctor or nurse directly involved in patient’s care	33	47.2
	Any nurse, doctor on call or clerical staff in the ward or unit where the patient is being managed	24	34.9
	Others (patient)	2	2.9

CV, critical value; SMS, short message service.

† , Respondents selected all that apply; hence, more than one response per respondent may be allowed as applicable in practice.

### Factors associated with the practise of critical value notification

The practice of CVN was not related to whether the laboratory was a private establishment or a public hospital laboratory (*p* = 0.39) nor the level of healthcare they serviced (secondary or tertiary), *p* = 0.89 (Table 4). Private laboratories were likelier to have separate paediatric CV lists than public hospital laboratories (*p* = 0.02) and to practise telephone notification than public or government hospital laboratories (*p <* 0.001). However, the practice of telephone notification was not related to the level of healthcare service, secondary or tertiary (*p* = 1.00).

**TABLE 4:** Factors associated with the practice of critical value notification of 134 surveyed laboratories across 25 states in Nigeria, October 2015 to December 2015.

Laboratory categories	Practice		*p*
	Yes	No	
**Practice of CVN**			
Government/public	39	42	0.39
Private	30	23	-
Secondary	8	8	0.89
Tertiary	61	57	-
**Paediatric CVN**			
Government/public	10	29	0.02
Private	16	14	-
Secondary	5	3	0.14
Tertiary	21	40	-
**Telephone notification**			
Government/public	11	28	< 0.001
Private	27	3	-
Secondary	4	4	1.00
Tertiary	34	27	-

Note: Compare government/public versus private and secondary versus tertiary.

CVN, critical value notification.

## Discussion

This survey showed remarkable variability in the policy and procedures of CVN across clinical laboratories in Nigeria, consistent with the findings in previously published surveys on CVs.^7,18,19,20,21^ Nearly half of the centres we studied did not consistently conduct CVN, and nearly two-thirds of those that did had neither a defined list of critical limits nor an existing guideline to govern CVN. This finding is worrisome, considering the importance of management of CVs in patient safety. Moreover, most of the centres surveyed serviced secondary and tertiary centres where specialist cases are managed, and a greater emphasis on timely results for prompt action is indicated. Although it is estimated that CVs comprise just about 2% of all laboratory results, laboratories are tasked with tracking this crucial part of the post-analytical stage.^18,20^ Even though the survey’s scope did not include the reasons for not using CVN, it is conceivable that some laboratory personnel may be unaware of the value of CVN. Our study’s findings differ from those of a survey conducted in Croatia, where less than 1% of the laboratories did not practise CVN.^18^ Other surveys reported between 2003 and 2011 in many high-income countries also show much higher rates.^19,20^ However, our result is consistent with reports in the last decade in some Southeast Asian and African countries, where substantial gaps in CVN practices were observed.^21,22,23,24^

Clinical laboratories are encouraged to provide a list of CVs for their users.^13,14^ Our findings show that the CV list was not readily available in many laboratories. There is a conspicuous unmet need for CVN, and laboratory professionals should lead in strengthening this aspect of the laboratory-clinical interface.^19^ The Australian consensus documents recommendations also specify that laboratories should, whenever possible, give separate lists for CVs to account for unique differences in patient groups and clinical contexts. The stratification may be by age, category of wards, inpatient or outpatient status.^7^ For many analytes, paediatric CVs differ considerably from adult values. Due to ongoing adaptations to extra-uterine environments, newborns and young children are especially prone to greater risk when alterations in biochemical composition in the body are not addressed promptly. Advisedly, it becomes imperative for critical limits to provide early enough warning to enable prompt action. In the United States, national surveys linked paediatric CVN with improved outcomes.^25,26^ Most laboratories that participated in the survey used similar critical values for paediatric and adult populations. The lack of separate lists for adult and paediatric CVs was more prominent in public hospitals than in private laboratories. This lack of separate lists for adult and paediatric CVs may reflect a limited understanding of CVN practice and a lack of input from clinicians and other relevant stakeholders.

Many professional organisations’ CV requirements rely heavily on published literature; however, clinicians are encouraged to contribute their professional experience.^7^ The findings in this study indicate that most CVN-practising laboratories assign critical limits to chosen analytes by coalescence of local stakeholder viewpoints, including those of clinical and laboratory staff and published literature. Nevertheless, several laboratories exploited published works without consensus from clinical stakeholders. However, most of the laboratories in our analysis lacked literature to support their critical limits. Schapkaitz et al. reported in 2014 that most laboratories in a survey of 36 clinical laboratories in South Africa have CV policies derived from local clinical opinion.^27^ The heterogeneity in the sourcing of CVs and the guiding policies that were observed in this study may impede the harmonisation of CV lists in Nigerian laboratories and has been previously noted as a challenge to CV management.^18^

Automatic repetition for critically abnormal results is common practice in clinical laboratories.^7^ Almost all of the laboratories in our survey repeated CVs before notification. Indeed, some authors have demonstrated that, at most, there is only a modest advantage of repeat tests with CVs over a single run.^28,29^ Besides, there are concerns about the impact of delayed results due to repeat testing on managing patients with critically abnormal results. Given that many laboratories lack the high-precision autoanalysers available in high-income countries, the advantage of repeat testing for abnormally crucial results may be lost in resource-constrained settings like Nigeria. To address the concern of turnaround time, laboratories should set up procedures whereby CVs are relayed immediately to the clinicians as preliminary results, while informing them of the final confirmatory result after repeat testing. This process will allow clinicians to make quick clinical judgments to determine if the test result is consistent with the patient’s condition and to prepare for the right course of action when the result is affirmed.^7^

We observed considerable variability in handling repeated CVs as most laboratories had no policy for handling repeat-tested CVs. After repeat testing, most laboratories reported the mean of the CVs, while others reported the most critical of the values generated or the least critical of the values. Since most laboratories do not have guiding policies, the decision was left at the discretion of individual laboratory operators. Although there is no consensus on which result to report, this decision may ultimately affect the frequency of test errors.^7^ Therefore, laboratories should determine the results to report based on the peculiarities of their analytical systems and audits based on patient outcomes.

Our survey also shows that, most commonly, senior laboratory staff, such as pathologists, pathologists-in-training, laboratory managers and senior laboratory scientists, reported CVs. In some laboratories, however, CVN is the bench technologist’s responsibility. In a similar study in Egypt, the hospital laboratory physician bore the responsibility for CVN, followed by the laboratory technician.^24^ It is likely that the reporting of critical values is related to the cadre of staff available in the laboratory and their numerical strength. Clinical laboratories in Nigeria, like many health institutions, are often understaffed.^30,31^ There may not be enough senior laboratory personnel to effectively cover all shifts. For effective CVN in the Nigerian laboratory setting, it is, therefore, imperative that all cadres of laboratory staff be suitably trained to recognise, authenticate and handle CVs. The need for adequate training, defined responsibilities, and procedures to notify CVs should been emphasised.^7,12^

The most common means of notification was physical dispatch. Almost half of the CVN-practising laboratories did not practise telephone notification. The definition of CVs requires prompt notification; thus, it is unlikely that physical dispatch would be able to achieve this. Telephone reporting is the cornerstone of rapid communication of results in laboratories in developed countries. In a Croatian survey, more than 90% of the laboratories practise telephone reporting, and in an Egyptian survey, the means of CVN was mainly by telephone.^18,24^ In the index survey, private laboratories were more likely than public or government hospital laboratories to use telephone notification. Since the majority of private laboratories are likely to view this as a crucial commercial service strategy to maintain their competitive edge, this may not come as a surprise. On the other hand, telephone service is not consistently available in many public hospital laboratories. Moreover, missing contact details for persons to inform in the event of a CV may be a plausible explanation.^32^ In many government hospitals, electronic medical records are unavailable, and test orders are placed manually. The hand-written paper requisition forms have only the name of the requesting physician or consultant in charge, with usually no provisions for telephone contacts in case of a CV. Many public hospitals in low- to middle-income countries grapple with the challenge of unreliable power supply, which may affect the use of technology in CVN.^32^ Therefore, in developing procedures for CVN in limited resource settings, this challenge must be considered and addressed. Laboratorians must liaise with clinicians and hospital management to emphasise the need to provide information for a reliable contact for CVN.

Timely reporting of critically abnormal results is central to effective CV management. In our survey, more than half of the laboratories did not have notification time limits for CVs. About a quarter of the laboratories reported CVs within 30 min of generating results, similar to a 2014 Croatian national survey finding.^18^ However, a 2019 study in Indonesia reported that about 99% of CVN was done within 7 min.^33^ Most laboratories notify the doctor or nurse directly involved in the patient’s care, similar to a Kuwait report in 2022, where ordering physicians and nurses were the most likely to receive a CV.^34^ However, most laboratories do not have mechanisms to deal with situations where the patient’s caregiver is unreachable, undermining the essence of notification of CVs for optimal patient care. Many guidelines recommend that laboratories develop structured algorithms describing actions to sustain communication when caregivers are unreachable. Alternative caregivers have been suggested in case of repeated failures; however, this will entail that the laboratory personnel are regularly furnished with contact information of the ordering clinicians and their surrogates.^7,35,36^

### Limitations

The number of respondents for the pre-test may not have been adequate, given the eventual sample size of the study. There may have been some selection bias since the question was completed by any senior laboratory staff deemed competent by the head of the laboratory to respond to the questions. The data on how long the responding staff had worked in the institution were not obtained. Therefore, some personnel may not be conversant with the CVN policies. Furthermore, advanced statistical analysis was not done to explore the reasons behind the practices of CVN by the laboratories surveyed, such as links with accreditation status. However, we have been able to describe the current practices across biochemical clinical laboratories across Nigeria.

### Conclusion

Our research has demonstrated that CVN practices are widely variable and often suboptimal in many clinical laboratories in Nigeria, which may be attributed to the absence of policies and defined procedures for CVN. The lack of national CVN recommendations or guidelines may exacerbate this situation. Training and capacity building in setting up, monitoring, and auditing CVNs are required to significantly enhance the practice of CVNs in Nigeria. This study’s findings have established a baseline for CVN that can be used to create a national consensus for CVN in Nigerian laboratories.
